# New frontiers to cure Alport syndrome: *COL4A3* and *COL4A5* gene editing in podocyte-lineage cells

**DOI:** 10.1038/s41431-019-0537-8

**Published:** 2019-11-21

**Authors:** Sergio Daga, Francesco Donati, Katia Capitani, Susanna Croci, Rossella Tita, Annarita Giliberti, Floriana Valentino, Elisa Benetti, Chiara Fallerini, Francesca Niccheri, Margherita Baldassarri, Maria Antonietta Mencarelli, Elisa Frullanti, Simone Furini, Silvestro Giovanni Conticello, Alessandra Renieri, Anna Maria Pinto

**Affiliations:** 1 Medical Genetics, University of Siena, Siena, Italy; 2 Core Research Laboratory, ISPRO, Florence, Italy; 3 Department of Medical Biotechnologies, University of Siena, Siena, Italy; 4 Genetica Medica, Azienda Ospedaliera Universitaria Senese, Siena, Italy

**Keywords:** Genetics research, Next-generation sequencing, Genomic engineering

## Abstract

Alport syndrome (AS) is an inherited genetic disorder characterized by range of alterations from glomerular basement membrane abnormalities up to end-stage renal disease. Pathogenic variants in the collagen α3, α4, and α5 encoding genes are causative both of the autosomal dominant and of the X-linked forms of AS. Podocytes are the only renal cells that are able to produce the COL(IV)a3-a4a5 heterotrimer. We have previously demonstrated how it is possible to isolate podocyte-lineage cells from urine of patients, providing an easily accessible cellular model closer to the podocytes’ physiological conditions. Taking advantage of disease-relevant cell lines, we employed a two-plasmid approach in order to achieve a beneficial and stable variant-specific correction using CRISPR/Cas9 genome editing. One plasmid carries a Donor DNA and a reporter system mCherry/GFP to track the activity of Cas9 in cells. The other plasmid carries a self-cleaving SpCas9 and the variant-specific sgRNA. We have analyzed two stable podocyte-lineage cell lines, harboring a variant in the X-linked *COL4A5* (p.(Gly624Asp)) and in the autosomal *COL4A3* gene (p.(Gly856Glu)). We have achieved reversion of variants greater than 40% with undesired insertions/deletions lower than 15%. Overall, we have demonstrated a new gene therapy approach directly on patients’ cells, key players of Alport pathogenesis, and we have reverted *COL4* causative variants towards the wild type state. These results, in combination with preclinical models, could open new frontiers in the management and the treatment of the disorder.

## Introduction

Alport syndrome (AS) (OMIM# 301050) is a clinically heterogeneous nephropathy caused by pathogenic variants in collagen IV genes (*COL4A3* [MIM# 120070] [Ref Seq NM_000091.4], *COL4A4* [MIM# 120131] [Ref Seq NM_000092.4], *COL4A5* [MIM# 303630] [Ref Seq NM_000495.4]) with a prevalence of 1:5.000 despite a very high variability among the populations [1]. The collagen α3α4α5(IV) heterotrimer represents an essential constituent of the mature Glomerular Basement Membrane (GBM) and is only produced by podocytes [2]. Podocytes are the key cellular component of the glomerular structure and can be isolated from Alport syndrome patients’ urine samples [3]. Typically, the disease, restricted to COL4, it is mainly characterized by a progressive ultrastructural damage of the GBM, which appears to be thickened and progressively delaminated. GBM disruption leads to severe clinical symptoms such as microhematuria, proteinuria, and an inexorable progression to End Stage Renal Disease (ESRD). Extrarenal manifestations, such as ocular (mono or bilateral hypoacusia) and visual manifestations (lenticonus and macular flecks) often worsen the clinical phenotype.

The disease is genetically heterogeneous, but the majority of AS kindreds show X-linked semi-dominant inheritance, due to pathogenic variants in the *COL4A5* gene located in the Xq22 region. In this form, males are more severely affected than females and usually reach end-stage renal disease before the age of 30 [4–6]. Females manifest a slow progression of the disease in 12% of reported cases, and some may develop ESRD later in life. The *COL4A3* gene, located in 2q36-37 head to head with *COL4A4*, is responsible for both autosomal recessive and dominant forms of AS [7–9]. The existence of an ADAS form is still debated. However, in the past decade, it has been shown that ADAS accounts for a statistically significant proportion of cases [7]. Furthermore, the existence of ADAS as a mode of inheritance distinct from TBMN has been supported by the employment of a Next Generation Sequencing (NGS) approach in patients presenting with GBM alterations overlapping with the abnormalities found in the XLAS and the ARAS form. In these patients, a second causative pathogenic variant in COL4 genes has not been found. Recently, Pescucci et al. delineated the overall clinical picture of ADAS, which is characterized by hematuria and proteinuria evolving towards ESRD with a mean age of ESRD onset of 55 years [10].

Starting from a bacterial immune system, the Clustered Regularly-Interspaced-Short Palindromic Repeat (CRISPR/Cas9) system has developed into a gene editing approach that could provide therapy for many rare genetic disorders [11]. In the years, several Cas9 nuclease variants have been discovered and characterized [12, 13]. The most widely used Cas9 variant derives from Streptococcus Pyogenes (spCas9) [14]. The CRISPR/Cas9 system is composed of two elements: the single-guide RNA (sgRNA) and the endonuclease Cas9. The sgRNA is able to guide the Cas9 to the genomic site of action where a highly precise double strand break (DSB) has to occur [14] following a Watson–Crick base pairing recognition [15]. DSBs induced by CRISPR/Cas9 are usually resolved through Non-Homologous End Joining (NHEJ), which can lead to creation of deletions and insertions. However, the precision of the DSB correction can be increased by a ‘repair template’ Donor DNA complementary to the genomic region of interest that can be used to perform neo-synthesis of wild type DNA [14].

The success of the CRISPR/Cas9 system is tightly correlated with the ability to introduce both components into mammalian cells. The CRISPR/Cas9 system has been proven to be a particularly dynamic and versatile tool over the last few years, and has been shown to be efficient in correcting in vitro a wide range of rare genetic disorders [16–18].

The few available therapies are restricted to the use of Renin–Angiotensin–Aldosterone (ARBs) inhibitors which aim to delay the progression of clinical symptoms. Up to date, considering that the pharmacological treatments (ARBs, ACEi) appear to be only effective in delaying the progression of clinical symptoms, the most successful treatment remains kidney transplantation. Despite their successful application in many cases of ESRD, transplants also present some limitations. They are restricted by the availability of a compatible donor, and by the necessity of a life-long immunosuppressive treatment in order to avoid Graft versus Host Disease (GVHD). Building on the availability of AS patients urine-derived podocyte-lineage cells we show that a tailored CRISPR/Cas9 system is capable of efficiently correcting COL4 causative variants in the disease-relevant cell line. This opens up the possibility of a novel therapeutic approach for Alport syndrome cure.

## Materials and methods

### Patient’s selection

Diagnosis of AS was established at the Medical Genetics Unit in Siena (Azienda Ospedaliera Universitaria Senese, AOUS). Families underwent genetic counseling and blood samples were collected in EDTA containing tubes for *COL4A3*, *COL4A4*, and *COL4A5* mutational analysis (next-generation sequencing and Sanger sequencing). Patients and healthy family members provided and signed a written informed consent at the Medical Genetics Unit of Azienda Ospedaliera Universitaria Senese, Siena, Italy for the use of DNA samples for diagnostic purposes and for urine samples collection with the purpose of isolating podocyte-lineage cells and characterizing intronic variants.

**Patient 1 (1212/18ats)**: The patient is affected by the semi-dominant X-linked form of Alport syndrome. She is a 23 years old woman, and has presented with persistent microhematuria since the age of three. During her childhood episodic gross hematuria occurred, accompanied by upper respiratory tract infections. At the time of the most recent genetic counseling, proteinuria was absent and renal function was preserved (creatinine: 0.6 mg/dL, eGFR: 127.6 mL/min). Audiological and ophthalmoscopic evaluations were reported as normal. Microhematuria and proteinuria were also reported in the father, who unfortunately was not available for genetic testing or counseling. NGS analysis performed on DNA from peripheral blood samples has revealed a *COL4A5* [MIM# 303630] ([MIM# 303630] [Ref Seq NM_000495.4]) heterozygous variant c.1871G>A (p.(Gly624Asp)) which affects the function of the gene. This variant, localized inside the interruption of the collagenic domain, is reported in association with a milder phenotype in males with an X-linked form of the disease. Clinical data and variants were submitted to the Leiden Open Variation Database LOVD, www.lovd.nl/COL4A5 (Individual ID #00245760).

**Patient 2 (3459/18ats)**: The patient is affected by the Autosomal Dominant form of Alport syndrome. She is a 41 year-old woman. She has presented with persistent microhematuria since age two, and developed proteinuria in her late thirties (1.6 g/24 h). A renal biopsy revealed GBM thinning and thickening. That combined with a lamina densa structure resulted in an Alport Syndrome diagnosis. Her audiological evaluation was normal, while ophthalmological evaluation showed astigmatism and myopia: both likely coincidental since they have not been previously reported as suggestive feature of AS. At the time of genetic counseling she was under therapy with Ramipril 10 mg/daily. Family history was suggestive of autosomal dominant AS. The younger brother, a 38 year-old male presented with microhematuria and proteinuria. He did not present with hematuria during infancy. Audiological and ophthalmoscopic evaluations were normal. The mother, a 71 year-old woman, reported the occurrence of microhematuria since age 32. Antihypertensive treatment was undertaken at the age of 68 to prevent progression to ESRD. Sensorineural hearing loss was detected by an audiological evaluation. NGS analysis revealed a *COL4A3* ([MIM# 120070] [Ref Seq NM_000091.4]) heterozygous variant c.2567G>A (p.(Gly856Glu)) which affects gene function. The same variant was also detected in both the affected proband’s mother and brother. Clinical data and variants were submitted to the Leiden Open Variation Database LOVD, www.lovd.nl/COL4A3 (Individual ID #00245757).

### Urine cell isolation

Urine from both patients one and two were processed within four hours of collection. Urine samples were centrifuged at 400 × *g* for 10 min. The pellet was washed with 10 ml of Washing Buffer and again centrifuged at 200 × *g* for 10 min. After the removal of the supernatant, the pellet was resuspended in 250 µl of DMEM/high glucose and Ham’s F12 Primary Medium and plated in gelatin treated culture dishes. Primary medium was added to the culture for the next three days (i.e., 24, 48, and 72 h after plating). Approximately 96 h after plating, one third of the medium was removed and 1 ml of RE/MC medium was added. The proliferation medium was changed daily, until two groups of small colonies were noticed: a first group of cells with a more regular appearance, smooth-edged contours, and cobblestone-like cell morphologies, and a second group, more randomly arranged with a higher proliferation rate (Fig. 2a Upper Right Panel). Cells were split around 9–12 days after plating.

### Preparation of the plasmids

Variant-specific sgRNAs were designed using the MIT CRISPR design tool (http://crispr.mit.edu). Both plasmids were cloned using a backbone from p.AAV2.1 obtained from TIGEM labs in Pozzuoli. The reporter plasmids were obtained by inserting the Human U6 promoter cassette for the expression of the sgRNA and the mCherry/GFP reporter system (Addgene #54322). Human U6 promoter was amplified by PCR using specific annealing primers (primers #1–2) from px330 (Feng Zhang’s Addgene plasmid #42230) with KOD Hot Start DNA Polymerase (Merck). The amplified product was cut and inserted into pAAV2.1, cutting the backbone using SacII and XhoI restriction enzymes at specific restriction sites. Oligonucleotides to create sgRNAs specific for the variants to target (#7–8/#9–10) were cloned after annealing and phosphorylation in the Bpil restriction site, under Human U6 promoter control. The mCherry/GFP reporter and the Donor DNA were synthetized (IDT, Coralville, Iowa, USA) and cloned into the U6 AAV vector in AflII and SacII restriction sites. The Donor templates (#11, #12) used to drive the correction of the target sequences were designed with 50 nt homology arms flanking the nucleotide to be corrected.

The autocleaving Cas9 plasmids were obtained by cloning the CMV promoter filled-in the sticky ends with AB Pfu polymerase (AB Analitica into its proper backbone. The backbone was then phosphorylated and inserted into NheI and NotI restriction sites. Then, the spCas9 was amplified by PCR (target-containing primers #3–4/#5–6) with KOD Hot Start DNA Polymerase (Merck), and cut and inserted in BamHI and SacII sites, under the control of the CMV Promoter.

The Oligos used for the cloning are listed in Table 1.

**Table 1:** List of Oligo used for cloning *COL4A3*/*COL4A5* plasmids

#1	Human U6 Fw	TAAACCGCGGGAGGGCCTATTTCCCATGAT
#2	Human U6 Rv	TAAACTCGAGGGTACCTCTAGAGCCATTTG
#3	*COL4A5 C*as9 Fw Primer	TAAAGGATCCCCTGGAGGGCCGAAATCAGGGGGATGG ACTATAAGGACCACGA
#4	*COL4A5* Cas9 Rv Primer	TAAACCGCGGCCTGGAGGGCCGAAATCAGGGGGTCA GCGAGCTCTAGGAATTC
#5	*COL4A3* Cas9 Fw Primer	TAAAGGATCCCCTGGAGAAACTGAATCACCAGGATGG ACTATAAGGACCACGA
#6	*COL4A3* Cas9 Rv Primer	TAAACCGCGGCCTGGAGAAACTGAATCACCAGGTCAG CGAGCTCTAGGAATTC
#7	*COL4A5* sgRNA Fw Oligo	CACCGCCTGGAGGGCCGAAATCAGG
#8	*COL4A5* sgRNA Rv Oligo	AAACCCTGATTTCGGCCCTCCAGGC
#9	*COL4A3* sgRNA Fw Oligo	CACCGCCTGGAGAAACTGAATCACC
#10	*COL4A3* sgRNA Rv Oligo	AAACGGTGATTCAGTTTCTCCAGGC
#11	COL4A5 Donor	CCCAGGTTTACCAGGCCTCCCAGGGAATATAGGGCCTATGGGTCCCCCTGgTTTCGGCCCTCCAGGCCCAGTAGGTGAAAAAGGCATACAAGGTGTGGCAG
#12	*COL4A3* Donor	AGACCCAGGAATTCCAGGCTTGGATAGATCAGGATTTCCTGGAGAAACTGgATCACCAGGAATTCCAGGTCATCAAGGTGAAATGGGACCACTGGGTCAA
#13	*COL4A5* sgRNA WT Fw Oligo	gtcgCCTGGAGGGCCGAAAaCAGGGGG
#14	*COL4A5* sgRNA WT Rv Oligo	cagaCCCCCTGgTTTCGGCCCTCCAGG
#15	*COL4A3* sgRNA WT Fw Oligo	gtcgCCTGGAGAAACTGgATCACCAGG
#16	*COL4A3* sgRNA WT Rv Oligo	cagaCCAGGTGATcCAGTTTCTCCAGG

Plasmids sequences have been submitted to Addgene Vector Database (https://www.addgene.org) with these accession numbers: for *COL4A3* Donor/Reporter (130279) and *COL4A3* self-cleavingCas9 (130281) for *COL4A5* Donor/Reporter (130280) and *COL4A5* self-cleavingCas9 (130282), respectively.

### Cell culture and transfections

HEK293T cells were cultured at 37 °C, 5% CO_2_, in Dulbecco’s modified eagle medium, (DMEM, EuroClone) supplemented with 10% fetal bovine serum (FBS; Carlo Erba), 2 mM l-glutamine (Carlo Erba), and 1 mM penicillin/streptomycin (Carlo Erba). Transient transfections were performed in 24-well plates using PEI (1 µg/µl) according to the manufacturer’s protocol. Hundred nanogram of reporter plasmid and 400 ng of Cas9 encoding plasmid were transfected. FACS analysis was performed 48 h after transfection.

Urine-derived podocyte-lineage cells were isolated from the urine of affected patients who harbor either a *COL4A3* ([MIM# 120070] [Ref Seq NM_000091.4]) (c.2567G>A p.(Gly856Glu)) and a *COL4A5* ([MIM# 303630] [Ref Seq NM_000495.4]) (c.1871G>A p.(Gly624Asp)) variant. 1 × 10^6^ cells were electroporated using the Neon® Transfection System (Thermo Fisher Scientific^®^, Waltham, Massachusetts, United States) in accordance with manufacturer’s protocol. Ten microgram of Reporter and 15 µg of Cas9 were co-transfected using the Neon System (1.150 pulse voltage [V], 20 pulse width [ms], 2 pulse number) according to the manufacturer’s protocol.

Untransfected cell population was used as negative control sample and cells electroporated with eGFP encoding plasmid (pAAV2.1_CMV_eGFP3) [19] alone were used as transfection positive control.

After transfection, cells were plated on 60 mm plate coated with 0.1% human gelatin (Merck Millipore^®^, Burlington, Massachusetts, United States), using RE/MC growth medium without penicillin/streptomycin.

### Isolation of mCherry^(+)^/GFP^(+)^ single cells using fluorescent activated cell sorting (FACS)

Cells were analyzed and sorted on a fluorescent-activated cell sorter FACSAria IIU using FACSDiva software version 8.0.1 (BD Biosciences). Cells were resuspended in PBS with a 2 mM EDTA. The cellular suspension was filtered through a 30 µm filcon filter (BD Biosciences). The urine-derived podocyte-lineage cells were sorted using a 70 μm nozzle and an event rate of 10,000/s.

EGFP and mCherry positive cells were quantified using a Cytoflex flow cytometer with blue (488 nm) and a yellow (560 nm) lasers, with a 530/30 (EGFP) and a 585/42 (mCherry) filter.

### DNA extraction

Total DNA was extracted from sorted cells using QIAMP DNa Micro Kit (QIAGEN^®^, Hilden, Germany) in accordance with manufacturer’s protocol. DNA from control cells was extracted starting from the same amount of cells. DNA was eluted into 25 µl of DNAse Free Water.

The concentration of RNA was determined by using a Qubit2.0 Spectrophotometer (Thermo Fisher Scientific^®^, Waltham, Massachusetts, United States).

### Ion torrent S5 sequencing

The Ion AmpliSeq 2.0 Library Kit (Life Technologies, Carlsbad, CA) was used for library preparation. The kit allowed us to obtain a barcoded library of 184 amplicons, corresponding to the 151 exons col *COL4A3/COL4A4/COL4A5* genes compatible with the ION S5 platform, according to the manufacturer’s protocol (https://assets.thermofisher.com/TFS-Assets/LSG/manuals/MAN0006735_AmpliSeq_DNA_RNA_LibPrep_UG.pdf).

Libraries were purified using Agencourt AMPure XP system and quantified using the Qubit^®^ dsDNA HS Assay Kit reagent (Invitrogen Corporation, Life Technologies), pooled at an equimolar ratio, annealed to carrier spheres (Ion Sphere Particles, Life Technologies) and clonally amplified by emulsion PCR (emPCR) using the Ion Chef system (Ion Chef, Life Technologies). Ion 510, 520, or 530 chips were loaded with the spheres carrying single stranded DNA templates and sequenced on the Ion Torrent S5 using the Ion S5 Sequencing kit, according to the manufacturer’s protocol.

Post-experiment analysis was conducted using the latest version (v5.8.0) of the data analysis software Torrent Suite (Life Technologies). A coverage assessment was performed using the ‘coverageAnalysis’ plug-in (v5.8.0.8) that gives information about the amplicon read coverage. Variants were called using the ‘variantCaller’ plug-in (v5.8.0.19).

## Results

### Editing strategy

**Fig. 1:**
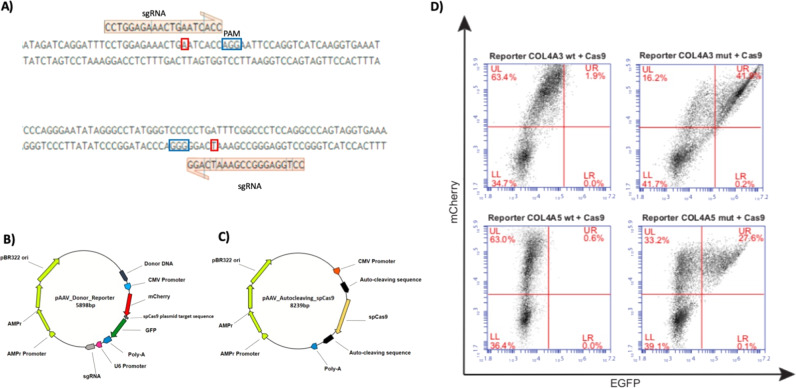
sgRNA identification and design and dual plasmid cloning strategy to achieved CRISPR/Cas9 correction. In the upper lane of panel **a**, the sgRNA is represented in red and blue: PAM in blue and in red the mutated nucleotide for the *COL4A5* CRISPR/Cas9 design. Below, the same design is reported for the *COL4A3* variant. Plasmid functionality is based on the cloning of two plasmids for CRISPR/Cas9 components delivery. In the first plasmid, the sgRNA is under the control of Human U6 Promoter (**b**), while the mCherry Red/Green Reporter System is under the control of a CMV Promoter. The Cas9 plasmid is cloned with spCas9 endonuclease gene under control of a CMV promoter flanked by two Self-Cleaving Cas9 for auto-inactivation (**c**). Representative FACS analysis on HEK293T cells after 48 h of transfection with reporter harboring the wild type sequence and mutated reporter, for *COL4A3* and *COL4A5* variants, respectively. The population of cells mCherry^+^/GFP^+^ is gated in the UR quadrant. The double fluorescence is present only in the cells co-transfected with Donor/Reporter plasmid with specific variant and spCas9 (41.9% and 27.6% for *COL4A3* and *COL4A5*, respectively) demonstrating the specificy of sgRNA (**d**)

Using the MIT CRISPR Design Tool, we selected a sgRNA that precisely targets the mutated *COL4A3* and *COL4A5* alleles (Fig. 1a). For the selected sgRNA an adjacent spCas9 PAM (N)GG is present on sense or antisense DNA strand. We have chosen to split the all-in-one plasmid into two distinctive plasmids, in a dual-vector approach, for contemporary delivery into the cells of the two components of CRISPR/Cas9 (Fig. 1b, c).

The Cas9 plasmid expresses the spCas9, under the control of the CMV promoter, and a targeting vector harboring variant-specific sgRNA, under the control of the Human U6 promoter. The Donor/Reporter plasmid contains a GFP coding sequence out of frame with an mCherry one. In between the coding sequences, there is a sequence target for the sgRNA. Induction of indels by Cas9 at the linker will place the GFP cds in frame with the mCherry and will cause the expression of an mCherry-GFP chimera, that will serve as a proxy to identify cells in which Cas9 has been active.

Moreover, in the Cas9 plasmid the variant-specific sgRNA with the PAM sequence has been cloned flanking the spCas9 itself, allowing Cas9 auto-cleaving and thus avoiding a prolonged expression in time and drastically reducing the off-targets cuts.

The mCherry/GFP reporter system includes a specific sequence, corresponding to the sgRNA + PAM, between the coding sequences (CDS) of two fluorescent proteins. When cells are transfected with the reporter plasmid alone, mCherry is transcribed, resulting in red fluorescence. In this condition GFP CDS is out-of-frame and it is not expressed. On the contrary when cells are co-transfected with the plasmid encoding spCas9, GFP expression is induced. Indeed, in this state, the sgRNA drives spCas9 to the sgRNA + PAM sequence between the two fluorescent proteins, and spCas9 cutting this sequence, reports GFP CDS in frame. As a consequence, an mCherry/GFP fusion protein is reconstituted and the two fluorescent proteins are co-expressed only in cells in which the spCas9 is active. Forty eight hours are necessary to induce mCherry and GFP expression, which is the time necessary for the spCas9 transcription and activation.

### Specificity of the sgRNA for the mutated allele

The double mCherry/GFP reporter plasmid allows us to view the cells in which the variant-specific sequence has been targeted by spCas9. Based on the same strategy, we then assessed whether the sgRNAs were able to recognize the mutated target, by inserting the wild type sequence between the two-fluorescence protein CDS. HEK293T cells transfected with the Donor/Reporter plasmid and spCas9 encoding plasmid yield a mCherry^(+)^/GFP^(+)^ population through FACS analysis after 48 h; the red fluorescence of co-transfected cells with the Donor/Reporter plasmid harboring the wild type sequence between mCherry and GFP, and spCas9 plasmid, marks the specificity of sgRNA only for the mutated target. Activation of spCas9 or not, depending on the co-transfection with Donor/Reporter plasmid mutated or wild type, is shown (Fig. 1d).

### Mutated podocyte-lineage cells transfection and editing

**Fig. 2:**
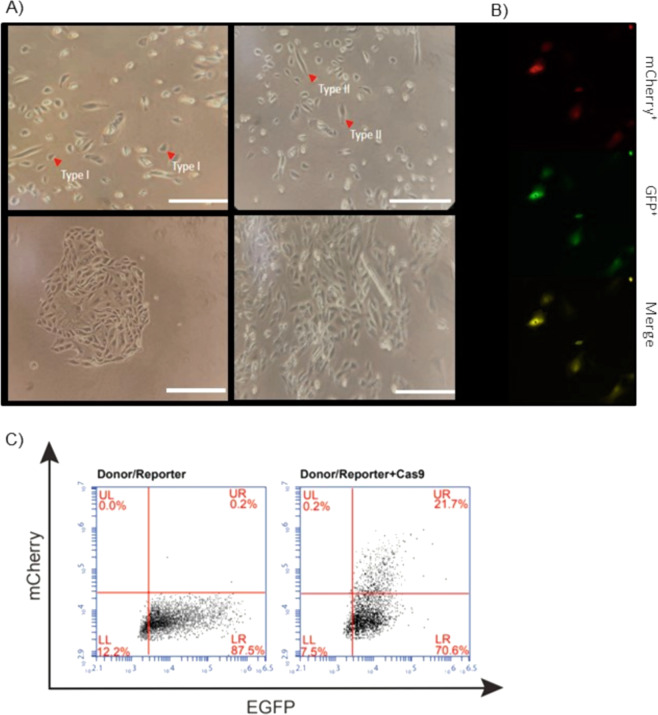
Transfection efficacy in urine-derived podocyte-lineage cells. **a** Type I (Upper left panel) and Type II (Upper right panel) urinary cell colonies (arrowheads) at day 4. At day seven the isolated cells start to aggregate in egg shape conformation (Bottom left panel). On the eighth day, cells starts to have clonal expansion and they can splitted (Bottom right panel) Scale bars, 400 μm. **b** Both plasmids were transfected on cells in clonal growth stage (Bottom panel) and mCherry and GFP fluorescence was observed in vivo through fluorescence microscope. **c** *Representative FACS analysis of GFP expressing*. Cells were transfected either with Donor/Reporter plasmid alone and sgRNA/Cas9 coupled with the Donor/Reporter plasmids. The cells were analyzed by FACS 48 h after transient transfection. The percentage of GFP + cells is indicated in the upper right quadrant (UR). At least three independent experiments were performed. The average population in the UR quadrant was 0.2% for Reporter alone; 21.7% for Cas9 + Donor/Reporter

To confirm the functionality of our strategy on urine-derived podocyte-lineage cells, (Fig. 2a, Bottom right panel) plasmids were tested by transient transfection.

Cells were transfected with the reporter plasmid alone, and also in combination with the plasmid encoding spCas9. Finally, they were analyzed by fluorescent microscopy after 48 h post-transfection. Urine-derived podocyte-lineage cells harboring a *COL4A5* ([MIM# 303630] [Ref Seq NM_000495.4]) variant (p.(Gly624Asp)) were transfected with both plasmids, whilst fluorescence was quantified 48 h post-transfection by fluorescence microscopy (Fig. 2b) and FACS analysis (Fig. 2c). Cell transfected with Donor/Reporter plasmid alone expressed mCherry but not GFP. On the contrary, among cells co-transfected with Donor/Reporter and spCas9 plasmids, a sizeable cell population expressed mCherry (35.5%), with 7.6% of these expressing also GFP. This result indicates a proper double-plasmid activation and the presence of a double-marked population that can be effectively recovered by means of FACS Sorter.

### Editing efficiency on mutated podocyte-lineage cells

To confirm the functionality of the system in AS podocyte-lineage cells we verified the correction of COL4 variants in patient-derived cells. Two stable podocyte cell lines carrying a specific variant in *COL4A5* ([MIM# 303630] [Ref Seq NM_000495.4]) c.1871G>A (p.(Gly624Asp)) and *COL4A3* ([MIM# 120070] [Ref Seq NM_000091.4]) c.2567G>A (p.(Gly856Glu)), respectively were transfected using our two-plasmid system harboring both a Cas9/sgRNA combination for a targeted dsDNA cut and a template Donor DNA for the neo-synthesis of a wild-type DNA fragment. The mCherry/GFP reporter was used to isolate cells where Cas9 had been active. Forty eight hours after transfection, cells were sorted to recover the doubly fluorescent ones (Red/Green). DNA was extracted and analyzed by deep sequencing.

For both the corrected variants, the IGV Visualization Software has been used to inspect both the snip out and the replacement of the mutated base and the indels events erroneously present in the neighboring sites.

FASTQ files from transfected and control samples were uploaded to the online analysis tool CasAnalyzer [20], in order to ascertain the percentage of Homology Directed Repair (HDR) achieved, considering a comparison range (R) of 15 nucleotides around the cut site.

**Fig. 3:**
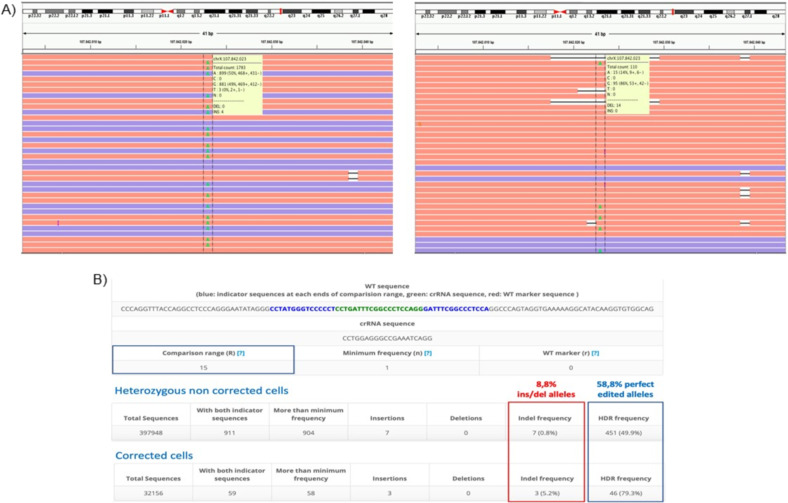
CasAnalyzer results on editing efficiency on *COL4A5* podocyte-lineage cells. CasAnalzyer Tool operating on the CRISPR/Cas9 treated sample and control has reported a HDR of 58.8% in patients’ mutated podocyte-lineage cells. IGV Visualization Software for the variant shows how there is a substantial loss of heterozygosity with the restoration of the wild-type base, confirming the functionality of the system

**Fig. 4:**
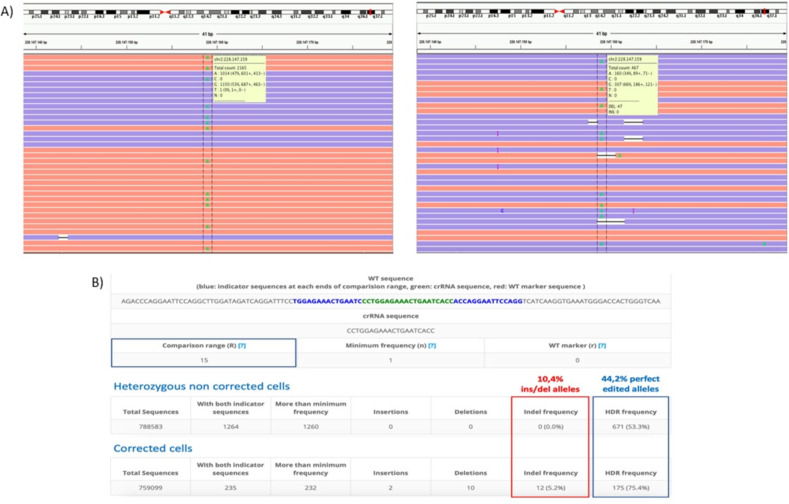
CasAnalyzer results on editing efficiency on *COL4A3* podocyte-lineage cells. The CasAnalzyer Tool performed on the CRISPR/Cas9 treated sample and control has reported a HDR of 44.2% in patients’ mutated podocyte-lineage cells. IGV Visualization Software for this variant also exhibited the loss of heterozygosity with the restoration of the wt base in *COL4A3* gene

Our system turned out to be extremely efficient (HDR of 58.8%) in reverting the causative variant in *COL4A5* and reaching an efficiency of HDR equal to 44.2% for *COL4A3* variant (Table 2). The results obtained from CasAnalyzer and IGV Software are reported in Fig. 3 for *COL4A5* (Fig. 3a, b) and Fig. 4 for *COL4A3* (Fig. 4a, b).

**Table 2:** CasAnalyzer results on the edited bases after CRISPR/Cas9 correction

Patient *n* (code)	Mutation	Sample	Total reads	HDR frequency	Ins/dels frequency *n* (%)	Wild Type reads = G (%)	Mutated reads = A (%)
1	*COL4A5* c.1871G>A (p.(Gly624Asp))	Edited cells	110	58,80%	10 (8,8%)	95 (86%)	14 (15%)
2	*COL4A3* c.2567G>A (p.(Gly856Glu))	Edited cells	467	44,20%	12 (10,4%)	307 (66%)	160 (34%)

### TP53 polymorphism characterization

It has recently been demonstrated that the reduced stability of *TP53* ([MIM# 191170] [Ref Seq NM_000546.4]) increases the rate of homologous recombination [21]. Therefore, we decided to characterize the functional genotype of *TP53*. The genotype of our patients at this locus demonstrated a heterozygous state (Arg/Pro) in both of them (Table 3) likely suggesting that polymorphism had an impact in modulating HDR rate.

**Table 3:** (p.(Pro72Arg)) genotype of TP53 in *COL4A3* and *COL4A5* mutated patients

Patient n (code)	Mutation	Pro72Arg genotype
1	*COL4A5* c.1871G>A (p.(Gly624Asp))	Arg/Pro
2	*COL4A3* c.2567G>A (p.(Gly856Glu))	Arg/Pro

## Discussion

Alport Syndrome is a clinically heterogeneous nephropathy caused by pathogenic variants in collagen IV genes. Today, no cure has been made available for this devastating disorder. Conventional pharmacological treatments have proved insufficient over the years, because they focus only on slowing down the development of clinical symptoms. The only effective treatment remains renal transplant, albeit with contingent difficulties in obtaining a compatible donor and subsequent immunosuppressive drug therapies that worsen the patient’s lifestyle. In addition, renal transplantation does not have an impact on affected extra-renal organs such as the eyes and ears. In this scenario, gene therapy aiming to correct the defect at the DNA level could be envisaged as revolutionary solution for an otherwise untreatable disorder.

The CRISPR/Cas9 technology in the past years, has emerged as an efficient solution to revert disease-causative variants. Using CRISPR, Xie et al. generated functional red blood cell precursors, ready for transplantation, from the fibroblast-derived iPSCs of a patient homozygous for β-thalassemia [18]. Two independent studies used CRISPR/Cas9 to correct alleles causing Duchenne muscular dystrophy in patient-derived iPSCs, further differentiated into skeletal muscle cells [16]. Rabai et al. have used CRISPR to correct heterozygous variants in the *DNM2* gene that causes the autosomal dominant form of centronuclear myopathies, a rare muscle disorder [22]. Bak and colleagues combined CRISPR/Cas9 technology with the use of rAAV6 to obtain a precise homologous recombination in human hematopoietic stem cells (HSCs) [23]. Ma et al. have performed the first CRISPR/Cas9 application in human preimplantation embryos, acting on *MYBPC3* variant and obtaining a high homology-directed repair efficiency by activation of an endogenous, germline-specific DNA repair response [24].

Over the last few years, a few CRISPR/Cas9 gene therapy-based trials have begun. In September 2018 the University of Pennsylvania, started a clinical trial based on engineering of T-cells from patients, using CRISPR to delete endogenous *TCR* and *PD-1* genes, giving the patients’ immune systems new tools to fight cancer cells (NCT03399448). Moreover, Vertex Pharmaceuticals Incorporated have proposed a phase 1/2 study to evaluate the safety and efficacy of autologous CRISPR-Cas9 Modified CD34^+^ Human Hematopoietic Stem and Progenitor Cells (hHSPCs) using CTX001 in subjects with severe sickle cell disease (NCT03745287). In February 2019, the University Hospital in Montpellier started to exploit epigenomic editing in Kabuki Syndrome as a therapeutic strategy to rescue the activity of *MLL4* (NCT03855631) taking advantage of a combination of the self-renewal potential of mesenchymal stem cells (MSCs) with CRISPR/Cas9 technology.

The objective difficulty in testing the CRISPR/Cas9 system on podocytes (the key cells affected in AS pathology) has always been a limit for preliminary in vitro studies aimed at effectively defining the *COL4* variant correction index. We have now gone beyond this technical limit, obtaining a cell population from the urine of affected patients in a completely non-invasive way [3]. This new cell population, the urine-derived podocyte-lineage cells, has provided us with an ad-hoc cellular model close to the natural podocytes physiological condition for each individual patient and each variant. Here, we have developed this novel tool to test two *COL4* variants, namely c.1871G>A (p.(Gly624Asp)) and c.2567G>A (p.(Gly856Glu)), in *COL4A5* and *COL4A3* respectively, mimicking two of the most frequent forms of hereditary transmission of the disease (XLAS and ADAS).

Here we report the correction of two disease-causative variants using an innovative self-inactivating dual-plasmid system through which homologous repair is driven by Cas9 induced DNA damage and a dsDNA donor fragment. The correction, as calculated by the CasAnalyzer tool, is very high, ranging from 44 to 58% in the *COL4A3* and *COL4A5* genes. These results along with the very low percentage of indels (8.8% for *COL4A5* and 10.4% for *COL4A3*) demonstrate that our strategy is highly efficient in inducing a dramatic genetic modification on the DNA. What is more: this approach is tailored to the specific variant as highlighted by the specificity experiments reported in Fig. 1d.

Furthermore, we hypothesized that the correction efficiency was also influenced by a functional polymorphism present in *TP53* gene ([MIM# 191170] [Ref Seq NM_000546.4]) where wild-type amino acid Proline is replaced by Arginine, affecting the stability of the protein [25]. *TP53* is a transcription factor involved in DNA damage repair. It has recently been shown that *TP53* is activated after dsDNA breaks induced by Cas9. Reduced stability of *TP53* increases the rate of HDR, promoting S phase transition of the cell cycle [21, 26]. The TP53Arg has been demonstrated to be less stable and transcriptionally less active than TP53Pro and this conformation of p53 induces a more frequent transition to S phase of the cell cycle promoting resolution of the dsDNA breaks through HDR events, in the presence of external Donor DNA [25, 26]. We thus hypothesized that the gene editing efficiency might be influenced by this polymorphism [c.215C>G (p.(Pro72Arg))]. Indeed, the presence of the same heterozygous genotype (Pro/Arg) in both patients may explain the relatively high HDR and low NHEJ (ins/del) reached, highlighting that p53 inhibition may modulate the efficiency of genome editing and suggesting that p53 function should be monitored when developing cell-based therapies that utilize CRISPR/Cas9.

One of the most challenging aspects of CRISPR/Cas9 human gene therapy is the in vivo distribution of plasmids for the activation of CRISPR/Cas9 machinery inside the affected cells. AAV provides one of the most suitable viral vectors to package, deliver, and express CRISPR components for targeted gene editing [27] given their ability to infect both dividing and non-dividing cells, their wide tropism and their low immunogenicity [15, 28]. Recent works have shown that AAV2 serotype is an efficient gene transfer vector to preferentially target renal cells [29], compared with AAV9 that instead is able to reach only tubular structure of the nephrons and thus AAV2 would be the most suitable system to deliver the developed CRISPR/Cas9 machinery to the podocytes, localized in the inner side of the Bowman’s capsule.

The results described in this work are a proof of concept for gene editing by CRISPR/Cas9 technology, and show that it is no longer an academic exercise, but an even more convincing reality. During the progression of AS, podocytes numbers substantially reduce due to structural defects that induce apoptotic processes and cell death. CRISPR/Cas9 gene therapy is likely to be efficient in the early stages of disease, where a podocyte reserve fraction is still present and can be ‘cured’, restoring their functionality and acting downstream in GBM.

Taking into account that AS is mainly a disorder of the kidney, but in some cases with involvement of eyes and ears, CRISPR/Cas9 gene therapy could be also proposed to treat visual and hearing difficulties.

CRISPR/Cas9 has already been developed and tested to treat visual disorders such as inherited retinal diseases. The eye is particularly suited to this treatment, given its isolated anatomical location. which allows an inoculation of the AAV virions directly in the affected organ [30, 31]. Thus, a localized CRISPR/Cas 9 injection could be taken into account for the treatment or even prevention of eye abnormalities if the systemic delivery would not allow a sufficient AAV9 concentration in this affected district. The same approach could be considered for the inner ear, where the variants in COL(IV) genes induce a detachment of the basal lamina with consequent loss of the hair cells that are mean to receive the auditory impulse. With the strong evidence that CRISPR/Cas9 system can efficiently target affected cells in vitro, testing the system on an in vivo model such as naturally occurring Navasota Dog (a *COL4A5* model) would be possible. We are therefore strongly convinced that AS will become a treatable disorder in the near future by a gene editing CRISPR/Cas9-based approach.
