# Correction: New frontiers to cure Alport syndrome: *COL4A3* and *COL4A5* gene editing in podocyte-lineage cells

**DOI:** 10.1038/s41431-023-01287-y

**Published:** 2023-01-18

**Authors:** Sergio Daga, Francesco Donati, Katia Capitani, Susanna Croci, Rossella Tita, Annarita Giliberti, Floriana Valentino, Elisa Benetti, Chiara Fallerini, Francesca Niccheri, Margherita Baldassarri, Maria Antonietta Mencarelli, Elisa Frullanti, Simone Furini, Silvestro Giovanni Conticello, Alessandra Renieri, Anna Maria Pinto

**Affiliations:** 1 Medical Genetics, University of Siena, Siena, Italy; 2 Core Research Laboratory, ISPRO, Florence, Italy; 3 Department of Medical Biotechnologies, University of Siena, Siena, Italy; 4 Genetica Medica, Azienda Ospedaliera Universitaria Senese, Siena, Italy

Correction to: *European Journal of Human Genetics* 10.1038/s41431-019-0537-8, published online 21 November 2019

The following acknowledgement was missing from this article.

Two of several authors of this publication are members of the “European Reference Network rare kidney diseases ERKNet”.

The original article has been corrected.

